# A PHENOMENOLOGICAL STUDY ON THE PERCEPTION OF FRAIL ELDERLY IN SUBJECTIVE HEALTH AWARENESS AND SUCCESSFUL AGING

**DOI:** 10.1093/geroni/igac059.2113

**Published:** 2022-12-20

**Authors:** Jisu Seo, Rhayun Song, Moonkyoung Park, Ahyun Ryu

**Affiliations:** Nursing, Daejeon, Taejon-jikhalsi, Republic of Korea; Chungnam National University, Daejeon, Taejon-jikhalsi, Republic of Korea; Chungnam National University, Daejeon, Taejon-jikhalsi, Republic of Korea; Chungnam National University, Daejeon, Taejon-jikhalsi, Republic of Korea

## Abstract

BackgroundFrailty is a multi-dimensional concept including physical, functional, and psychological aspects. The frail elderly have been assessed by various instruments, yet the classification of being frail is not often consistent with the perception of individuals. PurposeThis study aimed to explore the perception of frail older adults in subjective health awareness and successful aging. Methods9 participants who met the following inclusion criteria: older than 70 years, defined as frail status screened by frailty index, and signed the consent form. Data was collected through in-depth interview with recording, and evaluated by phenomenological approach of Colazzi. Results The content analysis on the perception of 9 participants on subjective health awareness yielded 7 theme-clusters and 3 categories: (a)Recognize frailty, (b)Vague and endless emotions, (c)Reflecting on the past and thinking about death. The participants described ‘recognizing frailty through senility’ as a subjective health awareness. The perception of participants on successful aging was structured 6 theme-clusters and 4 categories: (a)Sustainable health, (b)The goal of successful goodbye, (c)Responsibility for parenting, (d) The best wisdom that aging gives life. The participants described ‘living in the present and successfully parting with it’ as a successful aging. ConclusionThe participants who were categorized as frail status did not perceive themselves as frail. Many participants perceive being frail as natural process of aging, and their successful aging as sustainable health and successful goodbye. It is pertinent to understand the meaning of successful aging and subjective health awareness of frail older adults to provide effective intervention to help them.

